# Recommendations on the preparation of silicon solar cell samples for defect etching

**DOI:** 10.1016/j.mex.2022.101813

**Published:** 2022-08-08

**Authors:** Aleo Paolo Pacho, Markus Rinio

**Affiliations:** Department of Engineering and Physics, Karlstad University, Karlstad 651 88, Sweden

**Keywords:** Polishing, Secco etching, Grain boundaries, Dislocations, Etch pits, Photovoltaics, Multicrystalline silicon

## Abstract

Research on the structural defects of silicon such as grain boundaries and dislocations, their spatial distribution and how they impact the resulting solar cell performance often proceed by polishing the sample, etching to reveal the dislocations and grain boundaries, and then scanning the surface to image the defects and record their corresponding positions. While a lot of work has been devoted to developing appropriate etches and how to correlate the etch pits to cell performance, materials pertaining to preparation of samples for defect etching, which is a crucial step to ensure successful imaging and analysis, are limited. This work describes a method of polishing multicrystalline silicon solar cell samples in preparation for defect etching. The method described herein:

• Utilizes both mechanical and chemical mechanical polishing.

• Can be applied to both fabricated silicon solar cells and as-cut wafers.

## Graphical abstract

**Figure:**
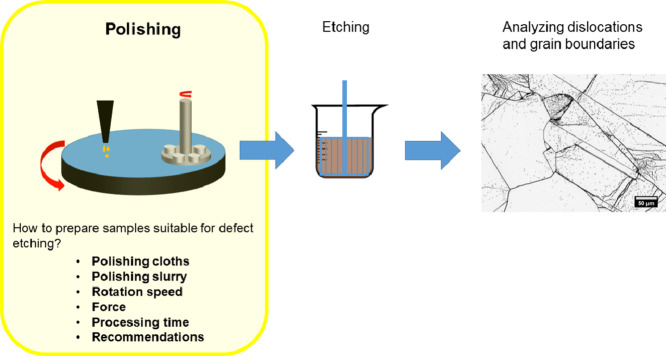

Specifications table

**Table:** 

Subject Area;	Materials Science
More specific subject area;	Defect engineering
Method name;	Polishing of multicrystalline silicon wafers
Name and reference of original method;	Metallographic polishing [1]
Resource availability;	Polishing machine SiC paper: 1200 and 2000 grit Polishing cloths Diamond suspensions with 9, 3 and 1 µm grain sizes Colloidal Silica slurry with 0.09 µm grain size, pH 11.9 Wax adhesive Optical microscope

## Method details

### Background

Defect etching is a technique used to reveal defects in silicon like dislocations and grain boundaries. Among the most utilized etches for this purpose is the Secco etch [2]. This has proven itself useful in several works that aim to quantify the impact of extended crystal defects on the performance of multicrystalline silicon (mc-Si) solar cells [3], [4], [5]. An important requirement for a successful etching is a clean and defect-free surface. Scratches, lapping marks and deformations on the sample, although barely visible under brightfield microscopy after polishing, will be exposed after etching. Such sample preparation artifacts can then severely complicate the image analysis of the etched samples.

Although polishing silicon is a standard process in the semiconductor industry, monocrystalline silicon wafers are typically used as substrates and are polished via chemical mechanical polishing (CMP) [6]. While this process produces surfaces planarized to a high degree of precision to meet the strict industry requirements, typical bench-top polishing machines used in metallographic laboratories are not specifically tailored for it. Furthermore, slurries used in CMP are selective [1,7]. They react with different types of materials and crystal orientations at different rates, constraining their use on multicrystalline materials.

Despite the importance of the sample preparation step prior to etching, references describing the actual procedure are limited to materials produced by polishing machine manufacturers, such as [8,9]. These rarely address silicon wafers and the more stringent surface quality requirements for submicron image analysis of individual defects. This work aims to fill this void by describing a typical process in preparing samples for Secco etching. The polishing method presented here is applicable for both full solar cells and as-cut wafers. It has been optimized for 25 mm x 25 mm samples.

### Cutting and mounting

**Fig. 1:**
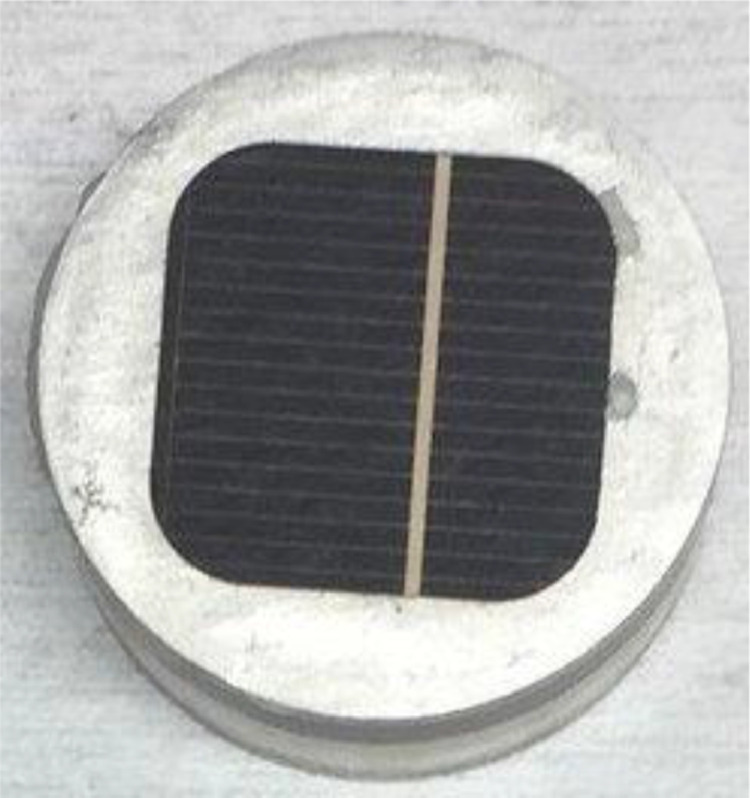
Photograph of a 25 mm x 25 mm mc-Si solar cell sample mounted for grinding and polishing.

Full size aluminum back surface field (Al-BSF) cells were laser cut into rounded squares of the aforementioned dimensions. Having the samples have rounded corners may lower the chance of edge chipping which may cause scratches. Proper care in cutting samples is important particularly for brittle samples prone to cracks like silicon wafers. The samples were mounted on aluminum sample holders using wax adhesive (Crystalbond 555). This is done by first placing the sample holder on a hot plate set at the melting point of the wax, which is around 50 °C for the wax used in this work. A thin uniform layer of the wax is then applied on the sample holder before placing the sample on top of it. A weight is placed on top of the sample to apply even pressure to push out air bubbles and make sure that the sample lies flat on the sample holder. The sample holder is then removed from the hot plate and allowed to cool down to room temperature for about 30 – 40 min. A photograph of the mounted sample is shown in Fig. 1. The polishing machine used in this work (Tegramin-30) is capable of polishing up to 6 such samples at a time. It is important to ensure that the sample is properly mounted before starting the actual grinding and polishing. Improperly mounted samples can lead to uneven grinding and polishing, and worse, leave hard to detect sample bits and fragments to the polishing cloths and therefore unwanted scratches on the samples. For our purposes, both sides of the samples were polished. After completing the polishing process for one side, the sample is unmounted, flipped and remounted so that the next side is facing up and ready to undergo the same polishing steps.

### Grinding and polishing steps

Table 1 shows the baseline recipe for polishing 25 mm x 25 mm mc-Si solar cell samples. Each row indicates a successive step in the polishing process, with each column showing the type of material for the polishing surface, the slurry used, the rotation speed of the polishing surface and the sample holder, the force exerted by the sample holder towards the polishing surface, and the processing time for each step. In all steps, the rotation of the polishing surface and the sample holder were in the same direction. While having the polishing cloth and the sample holder rotate at opposite rotations will increase the removal rate and therefore lower the step times, it also proved to be too aggressive and hard to control in our initial trials, often causing damage to the samples.

**Table 1:** Polishing recipe for 25 mm x 25 mm mc-Si samples. The brand names and manufacturers of the materials are listed inside parentheses.

Polishing Surface	Slurry/lubricant	disk rotation [rpm]	Sample rotation [rpm]	Force [N]	Time [min]
SiC 1200 grit (Struers)	Water	300	150	5	1
SiC 2000 grit (Struers)	Water	300	150	5	1
Taffeta woven wool (Struers MD-Mol)	Diamond suspension – 9 µm (Struers DiaPro Plan 9 µm)	150	150	10	3
Taffeta woven wool (Struers MD-Mol)	Diamond suspension – 3 µm (Struers DiaPro Mol B 3 µm)	150	150	10	5
Synthetic nap textile (Struers MD-Nap)	Diamond suspension – 1 µm (Struers DiaPro Nap B 1 µm)	150	150	10	5
Poromeric pad (Eminess Politex reg)	Colloidal Silica – 0.09 µm, pH 11.9 (Eminess Ultra-Sol 558)	150	150	20	15

**Fig. 2:**
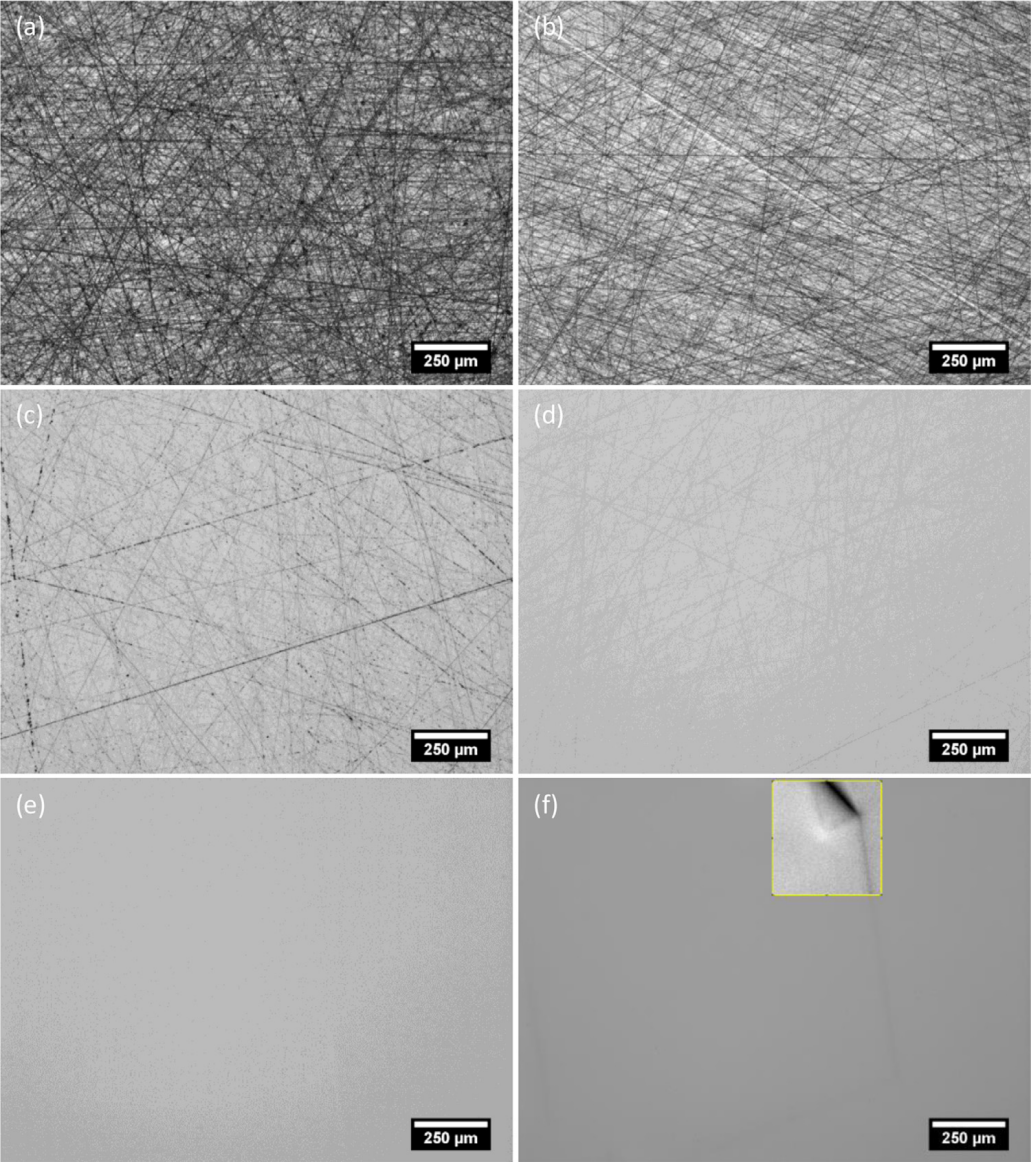
Optical micrographs of a sample region after each of the following polishing steps: (a) SiC 1200-grit grinding; (b) SiC 2000-grit grinding; (c) Diamond polishing – 9 µm; (d) Diamond polishing – 3 µm; (e) Diamond polishing – 1 µm; (f) Chemical mechanical polishing – colloidal silica. All images are taken under the same illumination conditions and exposure settings. The brightness and contrast of the highlighted region in (f) was adjusted to improve visibility.

The steps listed in Table 1 are meant to be performed in the order they appear. To achieve a surface free from scratches and deformations, successively finer abrasives are used. The first two grinding steps remove cell structures and surface features, ensuring that the sample surface is flat and ready for polishing. These are followed by mechanical polishing using diamond particles with grain sizes of 9 – 1 µm, and a final chemical mechanical polishing step using colloidal silica. The idea is to continuously remove material while removing scratches introduced by the previous step. It is therefore important that the sample and the polishing machine are cleaned thoroughly before proceeding to the next step. Any abrasive from a previous step or, even worse, large debris from samples, may cause deep scratches that cannot be removed by the finer abrasives of the next steps. It is also very important to inspect the samples after every step to ensure that the process went well and the resulting surface is as expected. It is in this regard that experience with polishing a particular material becomes extremely useful. Familiarity with how the sample should appear after a particular polishing step will help in determining how to proceed. To aid with this, optical micrographs of a mc-Si solar cell after the recommended polishing steps are shown in Fig. 2.

### Grinding

The grinding steps are the harshest among the steps, introducing deep scratches on the sample while removing all cell structures such as metal contacts and thin film coatings, as well as saw damage for the case of as-cut wafers. After grinding, the sample surface should be uniformly covered by a random scratch pattern. The presence of areas with obviously deeper or lighter scratches likely indicate improper mounting or worn out grinding papers. In such cases, it is best to remount the samples and repeat the grinding steps. Representative sample micrographs after grinding using SiC with grits 1200 and 2000 are shown in Fig. 2a and b respectively.

### Diamond polishing

The diamond suspensions used in this work (DiaPro) act as both lubricant and carrier of the abrasives. The dosing levels of the diamond suspensions were adjusted depending on the state of the polishing cloth, i.e. a new cloth requires more slurry for the first few uses, how often the cloths are used and how many samples are processed for a particular run. For the diamond polishing steps, the general rule is that the cloth must be damp but not soaking wet. Adding excessive amounts of polishing fluid reduces the polishing rate while very dry polishing cloths may cause surface blemishes and surface heating [1]. The typical consumption rate of the diamond suspensions is approximately 3 – 5 mL/min. In addition to the prescribed ‘run-in’ times for new polishing cloths, a quick ‘warm-up’ run for each step was performed by doing a 30 s run of the suspension on the textile without the samples at a higher flowrate (around 5 – 7 mL/min) and slower rotation (50 rpm). This is especially useful for cloths that are not regularly used. It also serves as a test run to verify that the polishing machine's components such as sample holder lock and suspension delivery systems are in proper order. A very noticeable change in the appearance of the sample surface can be seen after each of the diamond polishing steps. Specular reflection can be observed after the 3 µm polishing step but faint scratches are still present as shown in Fig. 2d. After the 1 µm diamond polishing step, the surface becomes mirror-like and appears to be scratch-free even under brightfield microscopy as shown in Fig. 2e. As noted previously, the sample should be monitored after every step. Cracks developing could be a sign of imminent sample breakage and must be taken into consideration before proceeding. Similarly, scratches that stand out from the uniform pattern, i.e. look deeper or wider than the majority of the scratches, are signs that something went wrong with the current step and may not be removable by the subsequent steps. Typical causes of such scratches are sample chipping, cloth damage and cloth contamination by larger abrasives. Depending on the severity, either the current or earlier steps should be repeated to remove the scratches. Note however that polishing times should be as short as possible to avoid polishing artifacts such as cracks and subsurface deformation and, especially for brittle materials such as silicon wafers, sample breakages. In any case, the sample must be inspected and evaluated whether repeating certain steps is necessary or if the damage is minimal and will not interfere with the analysis.

### Chemical mechanical polishing

**Fig. 3:**
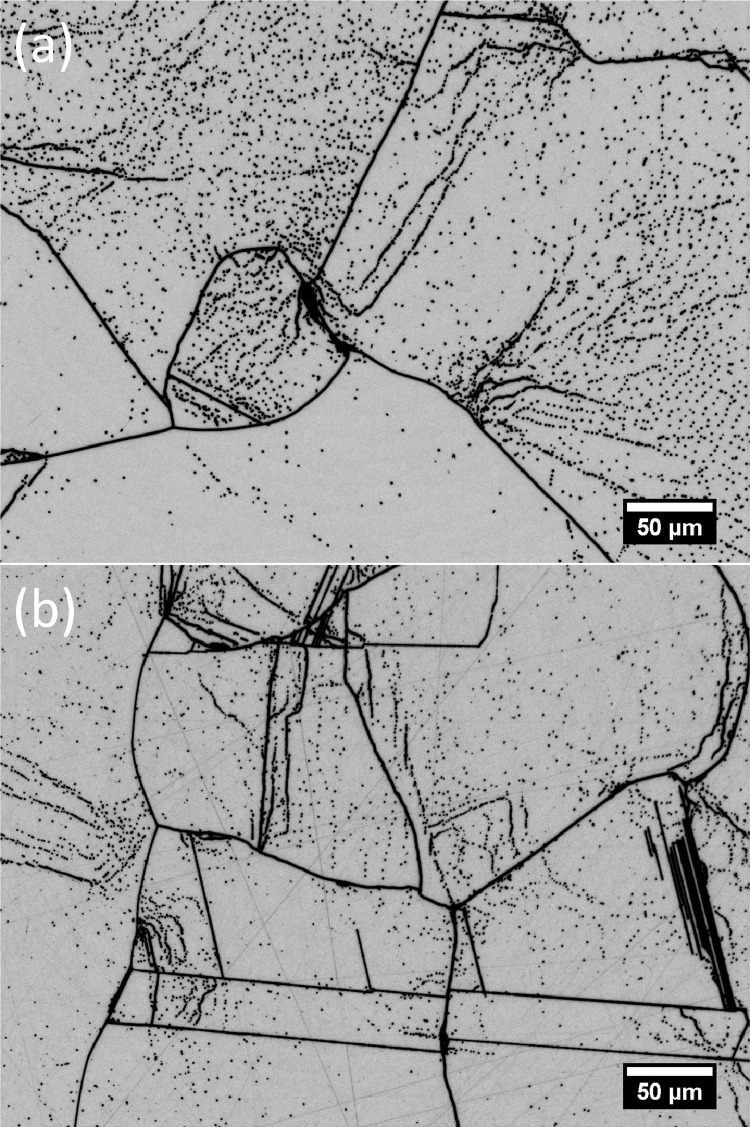
Images of samples after Secco etching (a) with and (b) without the chemical mechanical final polishing step. The brightness and contrast of the images were adjusted to improve visibility.

For final polishing of samples, the colloidal silica slurry Ultra-Sol 558 diluted at a ratio of 1:1 with deionized water was used at flowrates of around 40 – 50 mL/min. The material removal mechanism for this step is a combination of chemical reaction between the slurry and the surface, and mechanical abrasion by the silica in the slurry. This CMP step serves as final polishing to give a scratch and deformation free sample surface ready for defect etching. Although the surface already has a mirror-like finish and appears scratch-free after the last diamond polishing step even under a brightfield microscope, scratches still appear after Secco etching. Fig. 3 compares samples processed with and without the final CMP step after Secco etching.

**Fig. 4:**
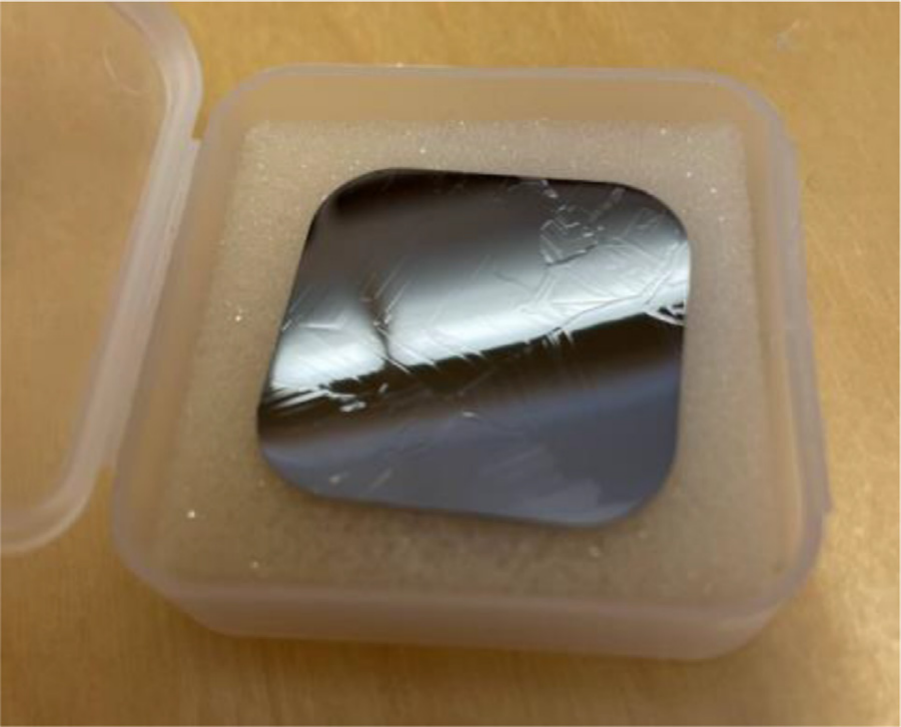
A photograph of a sample after the final CMP step.

As previously mentioned, some degree of selectivity is to be expected with CMP. This can be seen in Fig. 2f where some grains are faintly visible and appear to be higher than its surroundings. Such appearance is typical of the sample after this final polishing step. The surface is visibly specular just like after the 1 µm diamond polishing step but some grain structure is visible. This is shown in Fig. 4. This should be taken into consideration as it may affect the particular analysis to be performed on the sample. In our case, imaging the etched samples did not pose a problem since the autofocus system of the microscope was capable of coping with the variation in the topology of the surface.

Despite producing superior surface finish, CMP was only utilized as a final polishing step. This is because it proved to be considerably slower than diamond polishing in our initial tests. The mechanism behind this step affords damage free surfaces at the expense of lower removal rates. Initial tests showed that 1 h of polishing with colloidal silica and a polyurethane impregnated pad (SUBA 500) was not sufficient to achieve the required surface quality but often resulted in sample breakages.

### Method validation and some common pitfalls to avoid

**Fig. 5:**
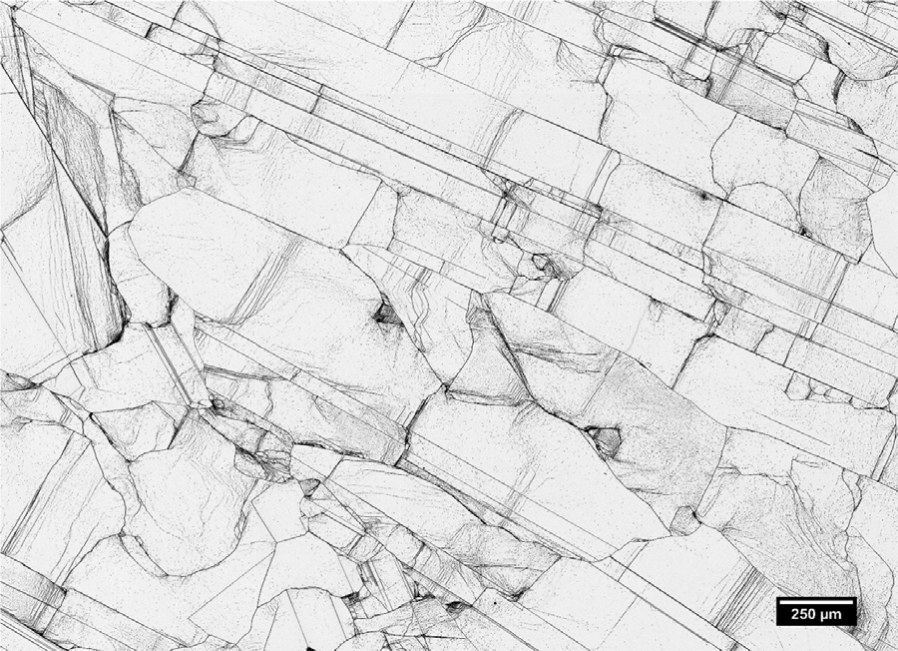
A stitched image of 7 × 7 images of a sample after polishing and etching. The brightness and contrast of the image was adjusted after performing shading correction to improve visibility.

The polishing method presented in this work has been used to prepare mc-Si solar cells for Secco etching. The etched samples were imaged using an optical microscope with a moveable stage at a resolution of about 0.34 µm/pixel. A typical result is presented in Fig. 5 where a grid of 7 × 7 individual microscope images are stitched together after performing shading correction. This method has been successfully employed in analyzing the images of etched samples and correlating them with internal quantum efficiency measurements using light beam induced current to quantify the impact of grain boundaries on the performance of mc-Si solar cells [10].

Proper sample preparation prior to defect etching is paramount to simpler and more efficient imaging and image analysis. Polishing a batch of samples, despite the short processing times listed in Table 1, can easily take up a whole working day since a significant amount of work must be done before processing and in between steps. The temptation to quicken the pace must be moderated by the fact that correcting for polishing artifacts like scratches in the etched sample, i.e. by manually deleting them in the images or adjusting the image analysis algorithm to disregard them, can be more time consuming and tedious. Mounting samples securely is the first and foremost protection for the samples. Cracks, edge chipping and sample breakages can be minimized with proper sample mounting. Having air bubbles or a significant gap between the sample and the sample holder is another important thing that is easy to overlook. Abrasives and polishing slurry can get trapped in between this gap and contaminate the cloth for the next polishing step, causing unintended scratches for that step despite the precautions taken to avoid cloth contamination. Cleaning the samples in between steps is crucial for a successful polishing and maintenance of polishing cloths. Similarly, skipping inspection of cloths before use and samples in between steps may seem to save time but will probably cause a larger inconvenience when dealing with the analysis of the etched samples. In the earlier and more damaging steps, deep scratches and uneven polishing is usually corrected only by repeating the grinding steps. With the more delicate steps, it must be carefully evaluated whether proceeding with the next steps and dealing with minor surface damages is acceptable to minimize the chance of sample breakages, or repeating earlier steps is necessary.

## Declaration of Competing Interest

The authors declare that they have no known competing financial interests or personal relationships that could have appeared to influence the work reported in this paper.

## Data Availability

Data will be made available on request.
